# Muscle texture features on preoperative MRI for diagnosis and assessment of severity of congenital muscular torticollis

**DOI:** 10.1186/s13018-024-04827-4

**Published:** 2024-06-20

**Authors:** Xin Qiu, Tianfeng Zhu, Zhenhui Zhao, Zhiwen Cui, Hansheng Deng, Shengping Tang, Leonardo Antonio Sechi, Gianfilippo Caggiari, Cailei Zhao, Zhu Xiong

**Affiliations:** 1 Shenzhen Children’s Hospital, Shenzhen, People’s Republic of China; 2 China Medical University, Shenyang, People’s Republic of China; 3 Nanshan District Medical Group Headquarters, Shenzhen, People’s Republic of China; 4 Orthopaedic Department, Sassari University Hospital, 07100 Sassari, Italy; 5 Department of Biomedical Sciences, University of Sassari, 07100 Sassari, Italy

**Keywords:** Congenital muscular torticollis, Radiomics, Muscle texture features, Magnetic resonance imaging, Diagnostic models

## Abstract

**Objectives:**

To develop an objective method based on texture analysis on MRI for diagnosis of congenital muscular torticollis (CMT).

**Material and methods:**

The T1- and T2-weighted imaging, Q-dixon, and T1-mapping MRI data of 38 children with CMT were retrospectively analyzed. The region of interest (ROI) was manually drawn at the level of the largest cross-sectional area of the SCM on the affected side. MaZda software was used to obtain the texture features of the T2WI sequences of the ROI in healthy and affected SCM. A radiomics diagnostic model based on muscle texture features was constructed using logistic regression analysis. Fatty infiltration grade was calculated by hematoxylin and eosin staining, and fibrosis ratio by Masson staining. Correlation between the MRI parameters and pathological indicators was analyzed.

**Results:**

There was positive correlation between fatty infiltration grade and mean value, standard deviation, and maximum value of the Q-dixon sequence of the affected SCM (correlation coefficients, 0.65, 0.59, and 0.58, respectively, *P* < 0.05).Three muscle texture features—S(2,2)SumAverg, S(3,3)SumVarnc, and T2WI extreme difference—were selected to construct the diagnostic model. The model showed significant diagnostic value for CMT (*P* < 0.05). The area under the curve of the multivariate conditional logistic regression model was 0.828 (95% confidence interval 0.735–0.922); the sensitivity was 0.684 and the specificity 0.868.

**Conclusion:**

The radiomics diagnostic model constructed using T2WI muscle texture features and MRI signal values appears to have good diagnostic efficiency. Q-dixon sequence can reflect the fatty infiltration grade of CMT.

**Supplementary Information:**

The online version contains supplementary material available at 10.1186/s13018-024-04827-4.

## Introduction

Congenital muscular torticollis (CMT) has an incidence ranging from less than 1–3.9% [1, 2]. Typically, CMT presents as a sternocleidomastoid muscle (SCM) mass 2 weeks after birth or with SCM contracture, with resulting limitation of head and neck tilt and rotation [3]. Pathological findings in affected muscle include fibrotic hyperplasia, fatty extracellular infiltration, and muscle fiber loss [4]. Early physical therapy is usually effective, but some children with SCM contracture may not significantly improve even after 6 months of physical therapy and will therefore need surgical treatment to avoid development of facial structural asymmetry and skull and spinal deformity [5, 6].

Currently, the diagnosis of CMT is mainly based on the medical history, physical examination, and ultrasound findings. Subjective interpretations may lead to misdiagnosis and missed diagnosis, and consequent delay in treatment. In addition, conventional ultrasonography cannot accurately identify the degree of fibrosis and fatty infiltration, and pathological examination can be performed only after surgery [4, 7]. Therefore, a reliable method for diagnosis of CMT and assessing its severity needs to be found.

A potentially useful modality is magnetic resonance imaging (MRI), which has the advantages of no radiation exposure, high soft tissue resolution, and multi-parameter imaging. Texture analysis (TA) of magnetic resonance images is an emerging field of radiomics in which quantitative or qualitative texture features from a region of interest (ROI) are used to clarify a lesion’s nature, treatment effect, and prognosis [8]. MRI and TA have been widely applied in diseases of the musculoskeletal system. For example, Mannil et al. [9] conducted TA on T2-weighted images of 62 patients with lumbar spinal stenosis and showed that some texture features can reflect the degree of paraspinal muscle fatty infiltration. Aurea et al. [10] identified three kinds of muscular dystrophy in mice by using TA on quantitative T2-weighted images. Other authors have shown that MRI combined with TA can help in differential diagnosis of various musculoskeletal diseases [11–13].

The aim of this study was to determine whether preoperative MRI of skeletal muscle and TA could provide objective criteria for diagnosis of CMT and assessment of its severity.

## Material and methods

### Ethical review statement

This study has been approved by the Medical Ethics Committee. The guardians of all participants in this study were thoroughly briefed about the study's content and objectives. Participation was voluntary, with informed consent forms duly signed by all parties.

### Study sample

The clinical and MRI data and postoperative histopathological results of 38 children with CMT who underwent surgery in our hospital from April 2019 to December 2019 were retrospectively analyzed.

### Methods

All 38 children underwent preoperative T1-weighted imaging (T1WI) and T2-weighted imaging (T2WI), and 20 children also underwent Q-dixon and T1-mapping. The researchers first manually outlined the ROI on the healthy and the affected sides and then used MaZda software to obtain the MRI signal values (maximum, minimum, mean, standard deviation) and muscle texture features on T2WI sequences. The end point event was defined as the diagnosis of CMT after the patient was examined by physical examination and ultrasonography. Logistic regression analysis was used to construct a radiomics diagnostic model based on muscle texture features and MRI signal values.

Pathological examination of skeletal muscle specimens was performed for all 38 patients. Fatty infiltration was graded on hematoxylin and eosin (HE)-stained sections and the degree of fibrosis on Masson-stained sections. See “Supplementary Material” for detailed methodology.

### Statistical analysis

**Fig. 1:**
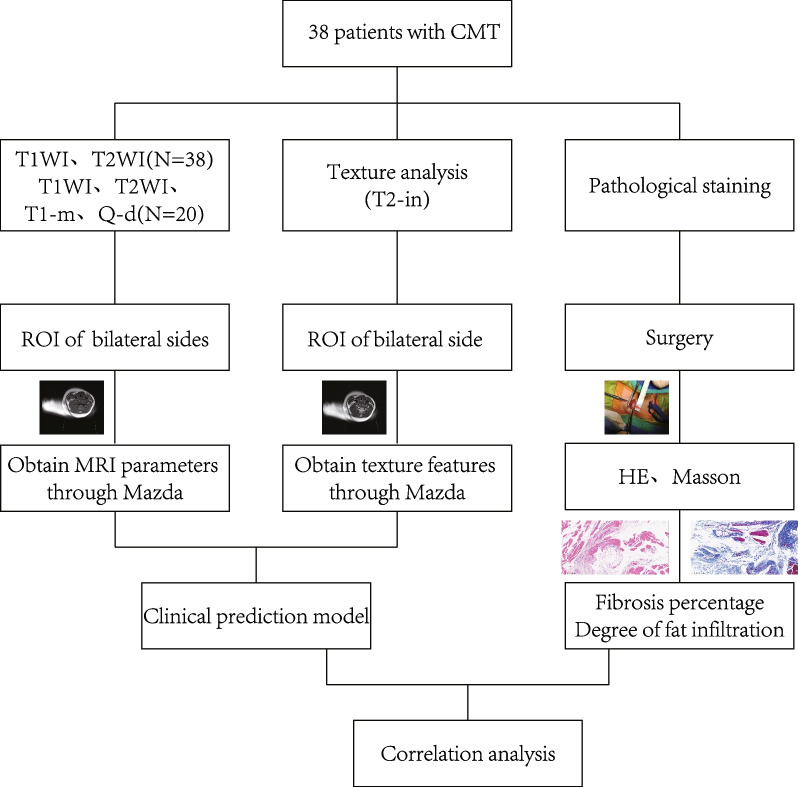
Overview of the method. The 38 patients underwent MRI, texture analysis, and pathological examinations. The region of interest on the healthy and affected sides were manually outlined in different sequences, and then MRI parameters of T1WI and T2WI, and the texture features on T2WI, were obtained using MaZda software. Sternocleidomastoid muscle tissue was obtained by surgery and stained with hematoxylin and eosin to assess degree of fatty infiltration and Masson stain to assess the degree of fibrosis. The diagnostic model for CMT was constructed using selected MRI parameters and texture features. Correlation between MRI parameters and pathology results were examined

MedCalc 22.0 software was used to remove redundant and irrelevant features by minimum redundancy maximum correlation. Data analysis was performed using R 4.1.0 software. Measurement data were expressed as means ± standard deviation and compared between groups using the paired *t*-test (for normally distributed data) or the Wilcoxon test (for non-normally distributed data). Enumeration data were expressed as number of cases (%) and compared between groups using the chi-square test. Lasso regression was performed using the glmnet package in R, and conditional logistic regression using the clogit package. The logistic regression model was constructed using the rms package. The Hosmer–Lemeshow test was used to test the model fit. Correlation between the indicators included in the diagnostic model, pathological indicators, and NMR signal values of Q-dixon and T1-mapping (Fig. 1) was analyzed using Pearson correlation analysis (for normally distributed indicators) or Spearman correlation analysis (for non-normally distributed indicators).

## Results

### Demographic and clinical features

The age of these 38 patients was 2.00 ± 0.52 years. The youngest patient was 5 months old, and the oldest was 10 years and 10 months old. There were 31 children below 3 years of age. CMT was on the left side in 14 patients and on the right side in 24 patients. The mean cross-sectional area of the SCM was significantly larger on the affected side than on the normal side (1.86 ± 0.69 cm^2^ vs. 1.17 ± 0.27cm^2^, *P* < 0.001; Table 1).

**Table 1:** Clinical features and data of 38 CMT patients

Variables	Statistics	*P* value
*Age*		
< 3 year	31 (81.6%)	
≥ 3 year	7 (18.4%)	
*Gender*		
Male	22 (57.9%)	
Female	16 (42.1%)	
*Affected side*		
Right	24 (63.2%)	
Left	14 (36.8%)	
*Cross-sectional area*		
Affected side	1.86 ± 0.69	< 0.001
Healthy side	1.17 ± 0.27	

### Ultrasonography findings

**Fig. 2:**
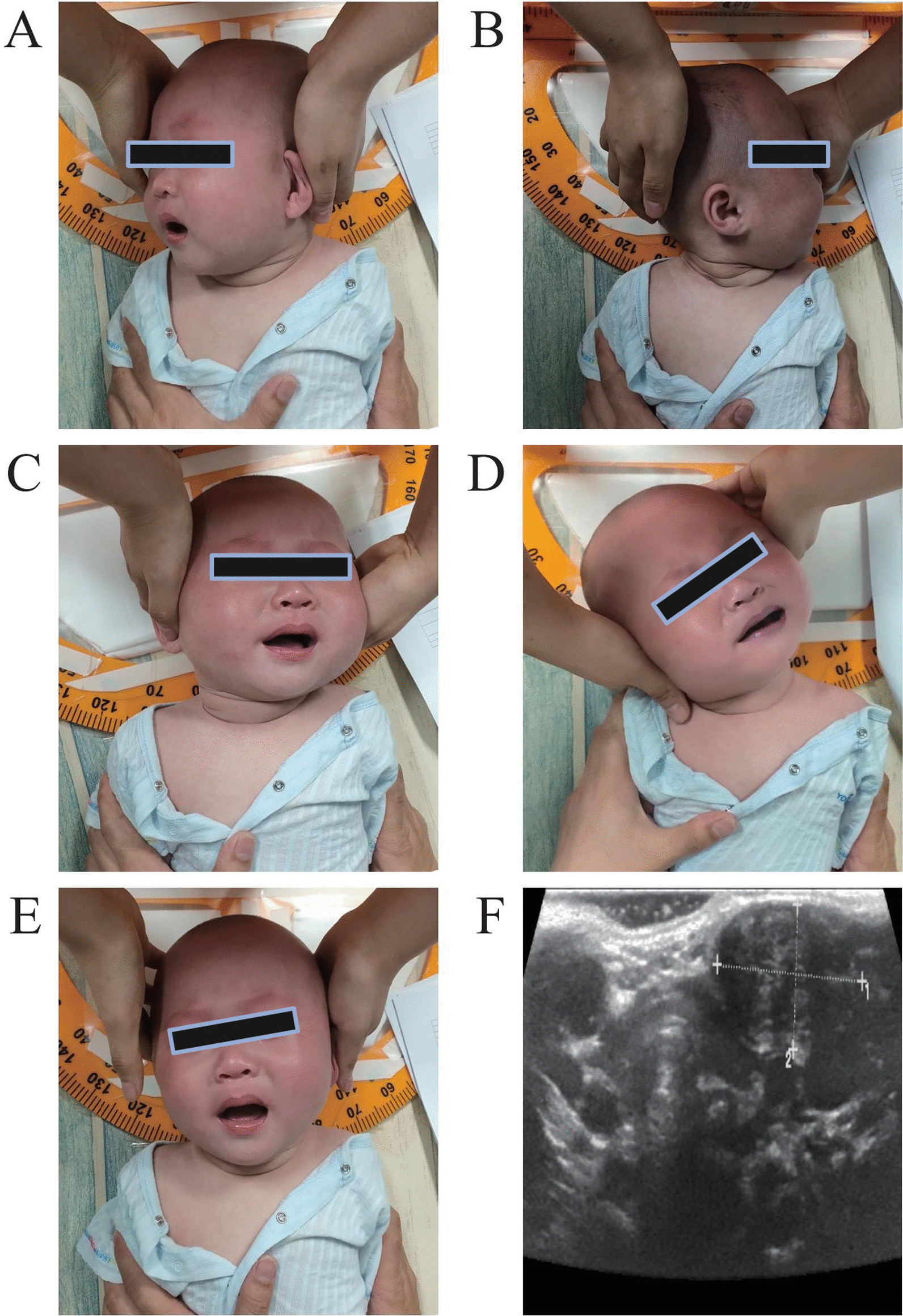
A patient with right congenital muscular torticollis. The patient has limited head rotation to the right but not to the left (**a**, **b**). There is limited head flexion to the left side but not to the right side (**c**, **d**). The patient’s head is tilted to the right under normal conditions (**e**). Ultrasound shows significantly thicker sternocleidomastoid muscle on the affected side than on the healthy side (**f**)

The affected SCM showed abnormal echoes with different degrees of hyperechoic areas (Fig. 2). A local mass-type lesion was seen in the SCM in 8 patients and uniform thickening of SCM in 30 patients. Among the 30 patients with uniform thickening of SCM, the maximum thickness of the muscle was significantly larger on the affected side than on the normal side (7.94 ± 2.36 mm vs. 4.85 ± 1.09 mm, *P* < 0.001). Among the 8 patients with local mass-type lesion, the mean volume of the mass was 7.184 ± 4.756 cm^3^ (Supplementary Table 1).

### MRI and muscle texture data

In T1WI of the 38 patients, the mean signal value in the ROI of the affected side is 196.40 ± 47.14, the mean signal value in the ROI of the healthy side is 258.47 ± 44.66, the mean signal value in the ROI was significantly lower on the affected side than on the healthy side (*P* < 0.001). In T2WI of the 38 patients, the mean signal value in the ROI of the affected side is 145.97 ± 51.61, the mean signal value in the ROI of the healthy side is 211.10 ± 43.65,the mean signal value in the ROI was significantly lower on the affected side than on the healthy side (*P* < 0.001).In T1 mapping of the 20 patients, the mean signal value in the ROI of the affected side is 1422.15 ± 136.79, the mean signal value in the ROI of the healthy side is 1671.05 ± 160.29,the mean signal value in the ROI was significantly lower on the affected side than on the healthy side (*P* < 0.001).

**Fig. 3:**
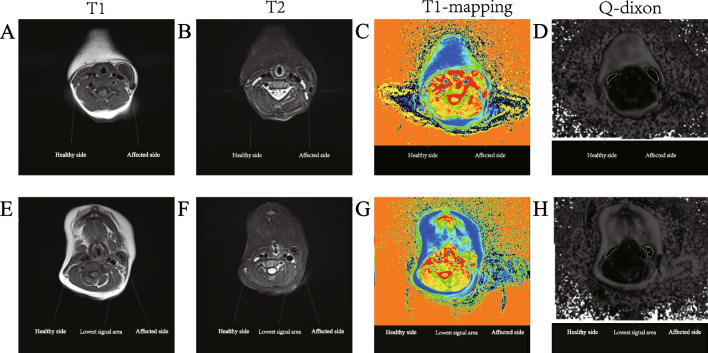
MRI imaging data and muscle texture data. The region of interest in the sternocleidomastoid of both sides was manually outlined, and T1WI, T2WI, T1-mapping, and Q-dixon sequences were obtained (**a**–**d**, respectively). The lowest signal area is seen in the affected SCM (**e**–**h**)

In addition, 20 children (53%) showed one or more T1WI and T2WI hypointensity areas in the ROIs of the affected SCMs (Fig. 3). The signal value in T1-mapping images was negatively correlated with the degree of fibrosis. The mean value of T1-mapping was significantly lower on the affected side than on the healthy side (*P* < 0.001; Supplementary Table 2). The acquired muscle texture features are divided into four categories: Histogram, GLCM, RLM, and Wavelet transform. (Supplementary Table 5).

### Histopathology

**Fig. 4:**
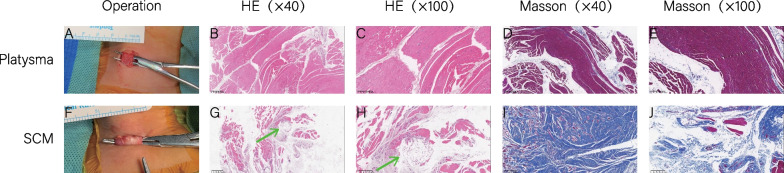
Histopathology results. Comparison of pathological examination results between platysma and affected sternocleidomastoid muscle. The platysma muscle is seen to be red during the operation (**a**). Hematoxylin and eosin (HE) staining shows only occasional adipocytes (**b**, **c**), and Masson staining shows only mild fibrosis (**d**, **e**). In contrast, the affected SCM has a white color (**f**). HE staining shows abnormal infiltration by adipocytes (green arrow; **g**, **h**). Masson staining shows that the fibrosis ratio is higher in the sternocleidomastoid muscle than in the platysma (**i**, **j**)

Masson staining showed that the percentage of fibrosis is 0.67 ± 0.05. HE staining showed fatty infiltration grade ranging from 0 to 4. While 10 children (26%) had grade 0 fatty infiltration, 28 (74%) had visible fatty infiltration (Fig. 4; Supplementary Table 3).

### Predictive model

After dimensionality reduction and modeling of statistical data, three indicators—S(2,2)SumAverg, S(3,3)SumVarnc, and T2WI extreme difference—were selected for incorporation into the multivariate conditional logistic regression model. The constructed model had significant diagnostic value for CMT (*P* < 0.05). High S(2,2)SumAverg was a significant risk factor for presence of CMT (OR = 1.27, 95% CI 1.07–1.50), while high S(3,3)SumVarnc indicated low probability of the disease (OR = 0.91, 95% CI 0.84–0.99; Table 2).

**Table 2:** Conditional logistic regression

	Odds ratio (95% CI)	Adj.odds ratio (95% CI)	*P* value
S(2,2)SumAverg	1.195 (1.066–1.34)	1.268 (1.074–1.497)	0.005
T2_max–min_	1.008 (1.002–1.015)	1.020 (1.006–1.035)	0.006
S(3,3)SumVarnc	0.97 (0.938–0.999)	0.912 (0.837–0.993)	0.033

**Fig. 5:**
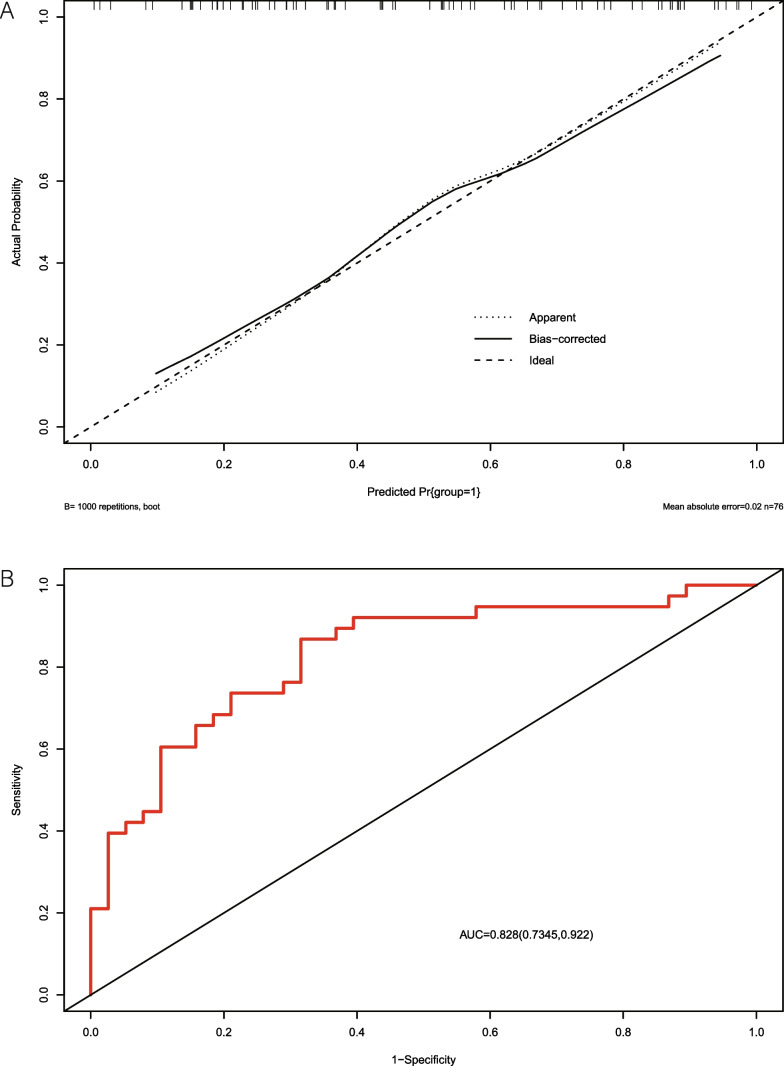
Results of Calibration curve of the model and ROC curve of the model. Calibration curve of the model (**a**). ROC curve of the model (**b**)

An unconditional multivariate logistic regression diagnostic model incorporating the above three indicators was constructed. This model was similar to those of the conditional multivariate logistic regression model (Table 3). The Hosmer–Lemeshow test showed that the model had a good fit (*χ*^2^ = 9.2752, *P* = 0.3196), indicating that the model was close to the actual identification model. The calibration curve for the Hosmer–Lemeshow test (Fig. 5A) showed a mean square error of 0.02.

**Table 3:** Unconditional logistic regression

	Odds ratio (95% CI)	Adj.odds ratio (95% CI)	*P* value
S(2,2)SumAverg	0.035 (1.046–1.114)	1.112 (1.038–1.209)	0.006
T2_max–min_	0.002 (1.001–1.005)	1.009 (1.003–1.015)	0.005
S(3,3)SumVarnc	0.017 (0.929–0.963)	0.943 (0.898–0.982)	0.009
(Intercept)		0.022 (0.002–0.189)	0.001

Receiver operating curve (ROC) analysis of the model (Fig. 5B) identified the optimal cutoff score of the model to be 0.366; the area under the curve (AUC) of this model was 0.8283 (95% confidence interval 0.7345–0.922), and the sensitivity and specificity were 0.6842 and 0.8684, respectively.

**Fig. 6:**
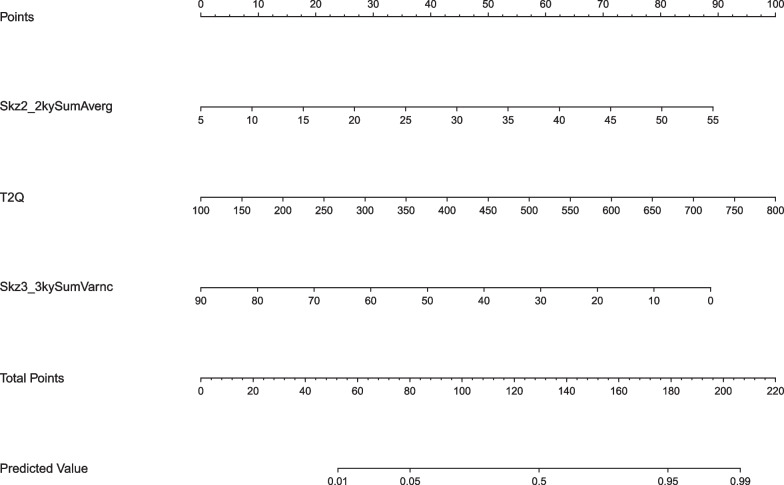
Line chart result

Finally, a predictive nomogram was constructed for clinical use (Fig. 6).

### Correlation analysis

The fatty infiltration grade on HE-stained specimens was positively correlated to the mean value, standard deviation, and maximum value of the Q-dixon sequence on the affected side (correlation coefficients were 0.65, 0.59, and 0.58, respectively, *P* < 0.05). The S(2,2)SumAverg and Q-dixon-Dixon sequence [(mean value of affected side − mean value of healthy side)/mean value of healthy side] were negatively correlated with fatty infiltration grade (correlation coefficient was − 0.57, *P* < 0.05). S(2,2)SumAverg was negatively correlated with the standard deviation of the T1-mapping sequence on the affected side (correlation coefficient was − 0.50, *P* < 0.05). S(3,3)SumVarnc was positively correlated to the minimum value of the T1-mapping sequence on the affected side (correlation coefficient was 0.47, *P* < 0.05) (Supplementary Table 4).

## Discussion

The diagnosis of CMT is currently based on subjective findings on physical examination and ultrasonography. There are still no quantitative indicators for diagnosis of CMT or preoperative evaluation of severity. We therefore constructed a radiomics-based diagnostic model using three MRI indicators: S(2,2)SumAverg, S(3,3)SumVarnc, and T2WI range. In addition, we showed that the Q-dixon sequence was correlated to the fatty infiltration grade. Thus, this study provides objective quantitative imaging indicators for better diagnosis of CMT and for formulating treatment plans.

We found differences in the MRI signal values of SCM between the healthy and the affected sides. The mean T1WI and T2WI signal values were smaller in the affected SCM than in the healthy SCM. The standard deviation of the T1WI and T2WI signals were larger in the affected SCM. HE staining and Masson staining showed that muscle fiber degeneration and muscle atrophy is common in CMT, with large amounts of hyperplastic collagen deposits appearing in the muscle interstitium. Previous studies have found that the T1-mapping signal value is inversely proportional to the degree of fibrosis. This was confirmed in our study, where the mean T1-mapping value was significantly smaller on the affected side than on the healthy side, indicating greater degree of fibrosis.

In addition, this study found that the SCM on the affected side also had the phenomenon of the lowest signal area. Among the 38 patients studied, 20 (53%) had one or more distinct T1WI and T2WI hypointense areas in the affected SCM. The area with the lowest signal was located in the ROI, suggesting that the most severe fibrosis occurs in the center of the SCM. Thus, when performing myolysis, the surgeon should thoroughly dissect the entire SCM and not just the surface or a part of the muscle. Extension of fibrosis to the surrounding normal muscle can only be avoided by complete dissection of the SCM. Previous studies have shown that areas with low signal have abnormal, disorganized connective tissue and interstitial fibrosis [14, 15]. Thus, the distribution of low-signal areas should be taken into consideration when formulating the surgical plan.

This study is the first to examine the use of TA in CMT diagnosis and to present a diagnostic model. TA has been found to be useful for diagnosis and assessment of severity of many other diseases. In patients with lumbar spinal stenosis, Mannil et al. [16] found that texture features extracted from T2-weighted images of paraspinal muscles correlated well with the results of traditional assessment tools such as Spinal Stenosis Measure, Roland–Morris Disability Questionnaire, and numeric rating scales.

In patients with CMT, fibrosis and fatty infiltration of SCM are the main pathological changes. We examined whether HE staining and Masson staining could reflect the degree of fibrosis and fatty infiltration. Previous studies have found that fibrosis is common in CMT, and that the degree of fibrosis is positively correlated with the severity of disease [4]. Gao et al. [17]used Sudan III staining to reveal fatty infiltration of SCM in CMT for the first time and reported that the fatty infiltration was in the form of clumps or cords. When skeletal muscle is chronically injured, muscle satellite cells are activated for repair. The activation and proliferation of muscle satellite cells is accompanied by an increase in lipid droplets, which decrease when the muscle satellite cells return to resting state [18]. Thus, fatty content in skeletal muscle may reflect the severity of disease [19]. In the present study, we also found good correlation between MRI findings and the degree of fibrosis and the degree of fatty infiltration as judged by HE and Masson staining.

The diagnostic model constructed using T2WI and TA parameters showed good diagnostic value for CMT. The conditional logistic regression model and the unconditional logistic regression model showed good diagnostic value. However, it should be noted that there is a risk of bias when an unconditional logistic regression model is applied to paired data. We also constructed a diagnostic nomogram for easy application in the clinic.

Correlation between pathological indicators and signal values of T1-mapping and Q-dixon was examined. Multiple signal values of the Q-dixon sequence were positively correlated with the grade of fatty infiltration, indicating that the Q-dixon technique could reliably indicate the degree of SCM fatty infiltration. S(2,2)SumAverg was negatively correlated with the standard deviation of the T1-mapping sequence, and S(3,3)SumVarnc was positively correlated with the minimum value of the T1-mapping sequence. Although these texture features did not show a direct correlation with fibrosis, T1 mapping might have potential value for identifying the degree of fibrosis in the SCM.

Literature review shows that the application of TA in the musculoskeletal system is relatively rare. So far, TA has mainly been applied in research on musculoskeletal tumors [20], osteoporosis [21], osteoarthritis [22], and cartilage structure [23]. Current evidence suggests that TA provides useful diagnostic information. Addition of TA to conventional imaging examination will improve the sensitivity and specificity of diagnosis. The combination can also be useful for monitoring disease progression and judging prognosis. A previous study on TA of the paraspinal muscles of healthy people revealed significant differences in fatty infiltration of the paraspinal muscles between different sexes and different anatomical locations [24], indicating that TA can identify subtle changes within muscles and potentially distinguish between physiological and pathological changes. In addition, TA may also be useful for disease risk prediction, as demonstrated by Thevenot et al. [25] who found that the combination of entropy, neck–shaft angle, and bone density had an AUC of 0.902 for predicting risk of femoral neck fracture.

This study has some limitations. First, this was a retrospective analysis of data of a small sample from a single center; bias is inevitable. The results of this study have to be confirmed in large well-designed studies. Second, the ROI was manually delineated; this may have impacted the results.

## Conclusion

To conclude, the radiomics diagnostic model constructed using T2WI texture features and signal values appears to be useful for diagnosis of congenital muscular torticollis. The Q-dixon sequence can reflect the degree of fatty infiltration in the affected SCM. TA may have potential value for identifying the degree of fibrosis. Future research should use machine learning approaches for construction of improved diagnostic models.

### Supplementary Information

Supplementary Material 1.

Supplementary Material 2.

Supplementary Material 3.

Supplementary Material 4.

Supplementary Material 5.

Supplementary Material 6.

## Data Availability

The datasets generated or analyzed during the study are available from the corresponding author on reasonable request.
