# Supplementary material for: Muscle texture features on preoperative MRI for diagnosis and assessment of severity of congenital muscular torticollis
Source: J Orthop Surg Res. 2024 Jun 20;19:367. doi: 10.1186/s13018-024-04827-4 (PMC11191279; doi:10.1186/s13018-024-04827-4)
Supplement: Supplementary file 1 — Supplementary Material 1. [file 13018_2024_4827_MOESM1_ESM.doc]

**Supplementary material**

**MRI data acquisition and analysis**

*MRI Data Acquisition Methods*

A Siemens Skyra 3.0T nuclear magnetic resonance imager (Germany) and a Head/Neck 20 MR Coil 3T phased array coil were used for conventional T1WI, T2WI, T1mapping and Q-dxion sequence coronal scans of the neck. All sequences were routinely shimmed before being scanned. Before the examination, the patient was given 10% chloral hydrate (0.5ml/kg), and the MRI scan was performed after the child was asleep. The patient is in the supine position. The MRI scan range is below the line between the eyes and above the mid-sternum. In addition, the scan range of MRI must encompass the sternocleidomastoid muscle on both the affected side and the unaffected side. All obtained MRI images were uploaded to the PACS workstation. On T1WI, T2WI, T1mapping, and Q-dxion sequences, the researchers selected the SCM of the healthy side and the affected side to be at the level with the largest cross-sectional area. At the level of the largest cross-sectional area, the researchers measured the maximum value, minimum value, average value, and standard deviation of the healthy side and the affected side of this level, respectively. In addition, the investigators measured the difference between the mean of the healthy side and the mean of the affected side, and the ratio of the difference between the mean of the healthy side and the mean of the affected side to the mean of the healthy side.

*The method of muscle texture data acquisition*

Export raw MRI data of all patients to DICOM image format. By Mazda software (Version 4.6, http://www.eletel.p.lodz.pl/programy/mazda/), ROIs were manually delineated in the images of the largest cross-sectional area of the SCM on the affected and healthy sides. The software automatically acquires muscle texture features, which are divided into four categories: Histogram, GLCM, RLM, and Wavelet transform, with a total of 290 sub-data. The above results were analyzed by 2 experienced diagnostic physicians in our research group using a blind method, and a consensus was reached after discussion on the results with dissent.

**Histopathological experiment**

The surgeon made a transverse incision 2 cm above the sternoclavicular joint on the affected side, cut the skin, carefully separated the surrounding soft tissues, and exposed the diseased SCM. The surgeon fixes the diseased tissue with large curved forceps, the clavicular head and sternal head are cut transversely, and 2-3cm of diseased tissue of the clavicular head and sternal head are cut. Immediately after the isolated tissue, it was fixed in 10% neutral formalin and routinely embedded in paraffin. The researchers performed Masson and HE staining on the pathological specimens and used a digital slide scanner (KF-PRO-005-EX/KFBIO) to scan the pathological images into images in KFB format. Under a 200x microscope, the researcher randomly selected ten non-overlapping fields of view for result interpretation and analyzed the following items:

a. Judging the grading of fat infiltration by HE staining: the researchers counted the number of adipocytes in each visual field and took the average value of 10 visual fields as the final counting result of adipocytes: no number was 0, 1-10 was Grade 1, 10-20 for grade 2, 20-50 for grade 3, >50 for grade 4.

b. The proportion of fibrosis area interpreted by Masson staining: the researchers used image analysis software (Image-pro plus version 6.0) to calculate the percentage of collagen fiber area/(collagen fiber + muscle fiber) area in each field view. The researchers took the average of 10 fields of view as the final area ratio of fibrosis.

**Screening and construction of model indicators**

The researchers described the overall situation of the study cohort, and the measurement data were expressed as mean ± standard deviation, median, and quartile. The count data are expressed by the number of cases (percentage). Based on the distribution characteristics of the data, the researchers used the t test or the rank sum test to compare the indicators of the healthy test and the affected side. The researchers explored relevant influencing factors according to the conditions of the affected side and the healthy side, and established a diagnostic model. The specific modeling method is as follows:

The researchers used minimum redundancy maximum correlation (mRMR) to remove redundant and irrelevant features and extracted 54 features. The researchers then select the features through the minimum absolute value convergence and selection operator (LASSO) regression, and obtain the lambda value corresponding to the minimum binomial deviation through ten-fold cross-validation, and keep the features where the coefficient is not zero, and finally get 32 variables. The researchers performed t-test or chi-square test on the 32 variables after dimension reduction. For data that conformed to normal distribution, the comparison between two groups was performed by paired t-test. For data that did not conform to non-normal distribution, the comparison between two groups was performed. The comparison between them was performed by Wilcoxon test.

Next, the researchers selected indicators with p<0.05, and performed conditional logistic regression on these indicators. The researchers used forward inclusion (P<0.1) and backward exit (P<0.01) methods to screen indicators, and constructed a multivariate conditional logistic regression model. At the same time, we performed unconditional logistic regression on the indicators included in the conditional logistic regression model.

Metrics for evaluating models

AIC value: AIC value can reflect the fitting situation of the model. The smaller the value, the better the model fit.

Calibration: The researchers used Bootstrap to perform 1000 resamples to evaluate the calibration of the model. The Calibration diagram and Hosmer-Lemeshow χ2 statistics are mainly used for reflection.

ROC curve: The prediction effect is evaluated through the ROC curve, and the area under the ROC curve is used as the evaluation index.

The researchers combined the above evaluation indicators to obtain the final diagnostic model.

**Correlation analysis**

The researchers conducted correlation analysis on the following three factors: ① three indicators of the model; ② MRI signal values of T1mapping and Q-dxion sequences; ③ grading of adipocyte infiltration and the proportion of fibrosis area. The researchers used the spearman or Pearson correlation test to screen out the relationship pairs with a correlation coefficient greater than 0.4 and p<0.05.
