# Supplementary material for: Muscle texture features on preoperative MRI for diagnosis and assessment of severity of congenital muscular torticollis
Source: J Orthop Surg Res. 2024 Jun 20;19:367. doi: 10.1186/s13018-024-04827-4 (PMC11191279; doi:10.1186/s13018-024-04827-4)
Supplement: Supplementary file 2 — Supplementary Material 2. [file 13018_2024_4827_MOESM2_ESM.docx]

| Supplementary Table 1.Ultrasonic data | | |
| --- | --- | --- |
| Variables | Statistics | P value |
| Uniform thickening N=30 | |  |
| Maximum thickness of affected side | 7.94±2.36 | ＜0.001 |
| Maximum thickness of healthy side | 4.85±1.09 |  |
| Local mass N=8 | |  |
| The volume of mass | 7.18±4.76 |  |
