# Supplementary material for: Muscle texture features on preoperative MRI for diagnosis and assessment of severity of congenital muscular torticollis
Source: J Orthop Surg Res. 2024 Jun 20;19:367. doi: 10.1186/s13018-024-04827-4 (PMC11191279; doi:10.1186/s13018-024-04827-4)
Supplement: Supplementary file 3 — Supplementary Material 3. [file 13018_2024_4827_MOESM3_ESM.docx]

| Supplementary Table 2.MRI parameters of the healthy side and the affected side in different sequences | | | | |
| --- | --- | --- | --- | --- |
|  |  | Affected side | Healthy side | P value |
| T1WI（N=38） | Minimum signal value | 85.61±45.81 | 182.37±48.03 | < 0.001 |
|  | Maximum signal value | 342.63±75.73 | 333.97±59.44 | = 0.672 |
|  | Mean signal value | 196.40±47.14 | 258.47±44.66 | < 0.001 |
|  | Standard deviation | 49.51±16.71 | 26.82±7.21 | < 0.001 |
| T2WI（N=38） | Minimum signal value | 29.74±20.77 | 103.87±49.37 | < 0.001 |
|  | Maximum signal value | 330.45±136.52 | 336.92±85.26 | = 0.805 |
|  | Mean signal value | 145.97±51.61 | 211.10±43.65 | < 0.001 |
|  | Standard deviation | 54.89±25.37 | 38.01±12.01 | = 0.002 |
| T1 mapping（N=20） | Minimum signal value | 802.50±243.67 | 1026.5±218.51 | = 0.004 |
|  | Maximum signal value | 2521.45±571.78 | 2659.95±560.31 | = 0.444 |
|  | Mean signal value | 1422.15±136.79 | 1671.05±160.29 | < 0.001 |
|  | Standard deviation | 276.55±99.67 | 277.30±154.17 | = 0.984 |
| Q-dixon（N=20） | Minimum signal value |  |  |  |
|  | Maximum signal value | 0.19±0.16 | 0.16±0.13 | = 0.508 |
|  | Mean signal value | 0.05±0.07 | 0.04±0.04 | =0.599 |
|  | Standard deviation | 0.05±0.05 | 0.04±0.03 | = 0.475 |
