# Supplementary material for: Muscle texture features on preoperative MRI for diagnosis and assessment of severity of congenital muscular torticollis
Source: J Orthop Surg Res. 2024 Jun 20;19:367. doi: 10.1186/s13018-024-04827-4 (PMC11191279; doi:10.1186/s13018-024-04827-4)
Supplement: Supplementary file 4 — Supplementary Material 4. [file 13018_2024_4827_MOESM4_ESM.docx]

| Supplementary Table 3. Results of HE and Masson staining | | | | | |
| --- | --- | --- | --- | --- | --- |
| Patient number | fibrosis ratio | Fat infiltration grading | Patient number | fibrosis ratio | Fat infiltration grading |
| 1 | 0.74 | 2 | 20 | 0.60 | 2 |
| 2 | 0.77 | 2 | 21 | 0.66 | 2 |
| 3 | 0.45 | 1 | 22 | 0.78 | 0 |
| 4 | 0.86 | 2 | 23 | 0.32 | 0 |
| 5 | 0.57 | 0 | 24 | 0.91 | 1 |
| 6 | 0.41 | 2 | 25 | 1.00 | 0 |
| 7 | 1.00 | 1 | 26 | 0.37 | 4 |
| 8 | 0.91 | 4 | 27 | 0.85 | 3 |
| 9 | 0.29 | 3 | 28 | 0.72 | 0 |
| 10 | 0.59 | 2 | 29 | 0.59 | 0 |
| 11 | 0.97 | 0 | 30 | 0.11 | 0 |
| 12 | 0.58 | 3 | 31 | 1.00 | 0 |
| 13 | 0.85 | 1 | 32 | 0.31 | 3 |
| 14 | 0.79 | 1 | 33 | 0.60 | 2 |
| 15 | 0.47 | 1 | 34 | 0.34 | 1 |
| 16 | 0.62 | 1 | 35 | 0.71 | 1 |
| 17 | 1.00 | 1 | 36 | 0.54 | 0 |
| 18 | 0.86 | 3 | 37 | 0.82 | 2 |
| 19 | 0.80 | 2 | 38 | 0.83 | 1 |
