# Supplementary material for: Muscle texture features on preoperative MRI for diagnosis and assessment of severity of congenital muscular torticollis
Source: J Orthop Surg Res. 2024 Jun 20;19:367. doi: 10.1186/s13018-024-04827-4 (PMC11191279; doi:10.1186/s13018-024-04827-4)
Supplement: Supplementary file 5 — Supplementary Material 5. [file 13018_2024_4827_MOESM5_ESM.docx]

| Supplementary Table 4.Results of correlation analysis | | | | |
| --- | --- | --- | --- | --- |
| Index 1 | Index 2 | Correlation method | coefficient of association | P value |
| S(2,2)SumAverg | {T1map}-Standard deviation of affected side | spearman | -0.501 | 0.026 |
| S(2,2)SumAverg | （DIXON）-Mean value of affected side①=F/（W+F）-The difference between the mean on the healthy side/Mean value of healthy side | spearman | -0.574 | 0.009 |
| S(3,3)SumVarnc | {T1map}-The minimum of the affected side | spearman | 0.474 | 0.035 |
| S(2,-2)SumVarnc | {T1map}-Mean value of affected side | pearson | 0.445 | 0.049 |
| S(2,-2)SumVarnc | T1map Mean value of affected side-The difference between the mean on the healthy side/Mean value of healthy side | pearson | -0.466 | 0.038 |
| Fat infiltration grading | （DIXON）-Maximum value of affected side | spearman | 0.581 | 0.007 |
| Fat infiltration grading | （DIXON）-Mean value of affected side①=F/（W+F） | spearman | 0.651 | 0.002 |
| Fat infiltration grading | （DIXON）-Standard deviation of affected side | spearman | 0.586 | 0.007 |
