# Supplementary material for: Muscle texture features on preoperative MRI for diagnosis and assessment of severity of congenital muscular torticollis
Source: J Orthop Surg Res. 2024 Jun 20;19:367. doi: 10.1186/s13018-024-04827-4 (PMC11191279; doi:10.1186/s13018-024-04827-4)
Supplement: Supplementary file 6 — Supplementary Material 6. [file 13018_2024_4827_MOESM6_ESM.docx]

| Supplementary Table 5. Categories of Muscle Textures | |
| --- | --- |
| Texture category | Texture feature |
| Histogram | Mean,Variance,Skewness,Kurtosis,Perc.01%,Perc.10%,Perc.50%,Perc.90%,Perc.99% |
| GLCM | AngScMom,Contrast,Correlat,SumOfSqs,InvDfMom,SumAverg,SumVarnc,SumEntrp,Entropy,DifVarnc,DifEntrp |
| RLM | RLNonUni,GLevNonU,LngREmph,ShrtREmp,Fraction |
| Wavelet transform | WavEnLL,WavEnLH,WavEnHL,WavEnHH |
